# Sino-Austrian High-Tech Acupuncture Network—Annual Report 2016

**DOI:** 10.3390/medicines4010011

**Published:** 2017-02-22

**Authors:** Gerhard Litscher

**Affiliations:** Research Unit for Complementary and Integrative Laser Medicine, Research Unit of Biomedical Engineering in Anesthesia and Intensive Care Medicine, and TCM Research Center Graz, Medical University of Graz, Auenbruggerplatz 29, 8036 Graz, Austria; gerhard.litscher@medunigraz.at; Tel.: +43-316-385-13907; Fax: +43-316-385-13908

**Keywords:** high-tech acupuncture, network, annual report, China, Austria

## Abstract

The Sino-Austrian High-Tech Acupuncture Research Network was founded in 2005 and has been growing ever since. The network comprises many partners from China and is highly involved in research and education activities. This report introduces the network’s activities in the year 2016.

The network was founded in 2005 by Prof. DDr. Gerhard Litscher from Medical University of Graz and comprises many partners from China (see Figure 1).

Within 2016, the Sino-Austrian High-Tech Acupuncture Network has grown very fast. Milestones and publications [1,2,3,4,5,6,7,8,9,10,11,12] from the year 2016 are listed chronologically below:

**12–19 March 2016:** Spring Seminar-Arlberg, Lech-Arlberg, Austria. Function Therapies. Society for Integrative Dental Medicine, CAM in Dentistry (Figure 2).

**1 April 2016:** Ohshiro Laser Therapy Best Paper Award, Tokyo, Japan, April, 2016 (Figure 3).

**14 May 2016:** WFAS-Education Working Committee Wuhan, China, 13 May 2016. (Figure 4) [6,8].

**20 May 2016:** 2016 IEEE Information Technology Networking. Electronic and Automation Control Conference (Figure 5).

**10–11 June 2016:** 11th International ISLA Congress for Medical Laser Applications, Beverungen, Germany (Figure 6).

**5 July 2016:** Joint research project with CACMS—China Academy of Chinese Medical Sciences (Figure 7).

**6 July 2016:** China Academy of Chinese Medical Sciences in Graz, Austria (Figure 8) [7].

Established in 1955, China Academy of Chinese Medical Sciences (hereafter abbreviated as CACMS) is China’s largest comprehensive research institute combining scientific research, medical treatment and teaching that is directly under the State Administration of TCM (traditional Chinese medicine). It boasts various disciplines, advanced equipment and great research strength and has under it 17 research institutes, six medical institutions, one graduate school, two branch schools, two pharmaceutical companies and publishing houses of ancient books on Chinese medical science. Besides, it is a founder of 13 kinds of academic journals on Chinese medical science.

What is especially worth mentioning is the achievement in artemisinin research, which provided a powerful weapon for humans against malaria and saved hundreds of thousands of lives, making tremendous contributions for human health, and thus CACMS were awarded by Lasker Medical Research Award and in 2015 the Nobel Prize in Medicine (Prof. Tu Youyou).

Acupuncture is one of the key disciplines of research of this renowned institution. Medical University of Graz (Prof. Gerhard Litscher) has a close cooperation with China Academy of Chinese Medical Sciences (with Prof. Yu Xiaochun) for more than 10 years, and also some activities with Tianjin University of Traditional Chinese Medicine on the topic of high-tech acupuncture research. Within the last years, more than 60 joint SCI/PubMed-listed publications have been published with CACMS alone. Prof. Litscher from Medical University of Graz is Visiting Professor at the Institute of Acupuncture and Moxibustion at CACMS.

**25 July 2016:** New Book-Heart Rate Variability and Acupuncture, Graz, Austria. Contact and order: gerhard.litscher@medunigraz.at (Figure 9).

**25 July 2016:** New Study—Laser Watch, Graz, Austria (Figure 10) [10].

**17 August 2016:** Hubei University of Chinese Medicine in Graz, Austria (Figure 11) [3].

Established in 1958, the Hubei University of Chinese Medicine has three secondary schools, four affiliated hospitals, four State-Level (highest level) Medical Research Centers and 10 Research Institutions. The university occupies 0.65 km^2^ with a total construction of about 470,000 m^2^. The university has 15 departments, 17 specialties for bachelor’s degree and 9 specialties for professional training, 19 specialties for master’s degree and 12 specialties for doctor degree; more than 60 bases for clinical practice, including 6 affiliated hospitals, 21 State-Level, Province-Level or College-Level laboratories for teaching. At present the university has about 15,000 students.

Acupuncture is one of the key disciplines of research of this renowned university. The Medical University of Graz (Prof. Gerhard Litscher) has a close cooperation with Hubei University of Chinese Medicine (with Prof. Wang Hua and Prof. Liang Fengxia) on the topic of high-tech acupuncture research. Within the last years, joint SCI/PubMed-listed publications have been published. Prof. Litscher from the Medical University of Graz is Visiting Professor at Hubei University of Chinese Medicine and at the Hubei Provincial Collaborative Innovation Center of Preventive Treatment by Acupuncture and Moxibustion (Director: Prof. Wang Hua).

**5 September 2016:** Acupuncture Congress—Experts. Timmendorfer Strand, Germany (Figure 12).

**16–17 September 2016:** International Laser Medical Congress, Sandvika, Norway (Figure 13).

**23 September 2016:** Meeting of Sino-Austrian auricular acupuncture leaders, Beijing, China (Figure 14) [2].

**23 September 2016:** Meeting-Hospital of Acupuncture and Moxibustion of China Academy of Chinese Medical Sciences (CACMS), Beijing, China (Figure 15).

**23 September 2016:** Editor’s Meet in Beijing-*Medicines*, Beijing, China, G. Litscher: Editor-in-chief of *Medicines* (Figure 16).

**24 September 2016:** Meeting at Beijing Hospital of TCM affiliated to Capital Medical University Beijing, China (Figure 17).

**26 September 2016:** Meeting at Tong Ren Hospital affiliated to Capital Medical University Beijing, China (Figure 18).

**26 September 2016:** China Academy of Chinese Medical Sciences, Institute of Acupuncture and Moxibustion, Beijing, China (Figure 19).

**27 September 2016:** Annual report meeting of guest professor at Hubei University of Chinese Medicine, Wuhan, China (Figure 20).

**27 September 2016:** Lecture at Hubei University of Chinese Medicine, Wuhan, China (Figure 21).

**28 September 2016:** WFAS-Education Working Committee, Wuhan, China (Figure 22).

**30 September 2016:** Discussion about “Phytopharm 2017” and 10th Anniversary of the TCM Research Center Graz in 2–5 July 2017, Graz, Austria (Figure 23).

**15–16 October 2016:** Laser Workshop, Munich, Germany (Figure 24).

**21–22 October:** TCM Symposium, Krems, Austria (Figure 25).

**9 November 2016:** Peking University-Health Science Center. Eurasia Pacific Uninet (EPU) (Figure 26).

**9–20 November:** USTB-University of Science and Technology Beijing, China (Figure 27).

**12 November 2016:** People’s Liberation Army General Hospital Beijing, China (Figure 28).

**14 November 2016:** Lecture at Tong Ren Hospital affiliated to Capital Medical University, Department of Anesthesiology, Beijing, China (Figure 29).

**16–19 November 2016:** 3rd Annual World Congress High-Tech Acupuncture and Integrative Medicine and 1st Annual World Congress Modern Chinese Medicine, Nanjing, China.

Venue: Nanjing International Youth Convention Center, China.

Congress Chair of both Congresses: Gerhard Litscher, Medical University of Graz, Austria, Europe. Congress Co-chair: Lu Wang, Medical University of Graz, Austria, Europe. Executive Chair: Xiaodan Mei, BIT, Dalian, China.

Experts from many different countries and regions from all over the world have presented their research results. Altogether, 70 speakers have presented their lectures and workshops during the three days.

Opening Ceremony: Three Nobel Prize winners (Figure 30) at the World Congress (Figure 31).

**18 November 2016:** Discussion about tradition and innovation of auricular acupuncture for the Singapore conference in August 2017, Nanjing, China (Figure 32).

**3 December 2016:** Traditional Chinese Medicine—Master of Science Medical University of Vienna, Austria (Figure 33).

**15 December 2016:** Meeting of Eurasia Pacific Uninet (EPU) Scholars. University of Veterinary Medicine, Vienna, Austria (Figure 34).

## Figures and Tables

**Figure 1:**
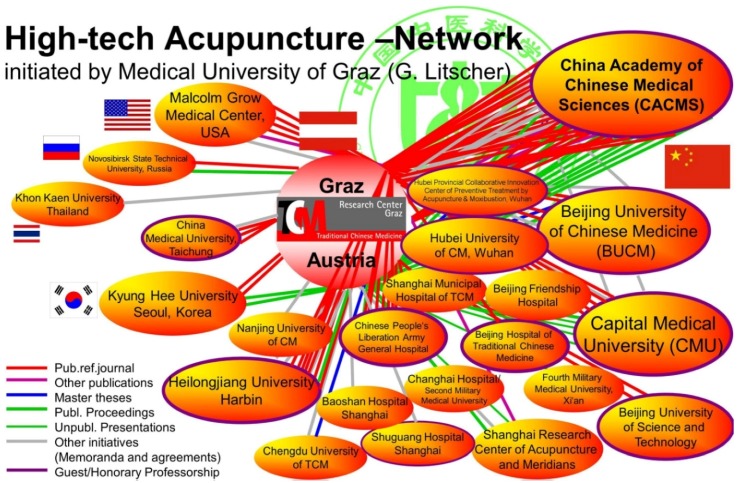
High-tech acupuncture network partners and scientific information.

**Figure 2:**
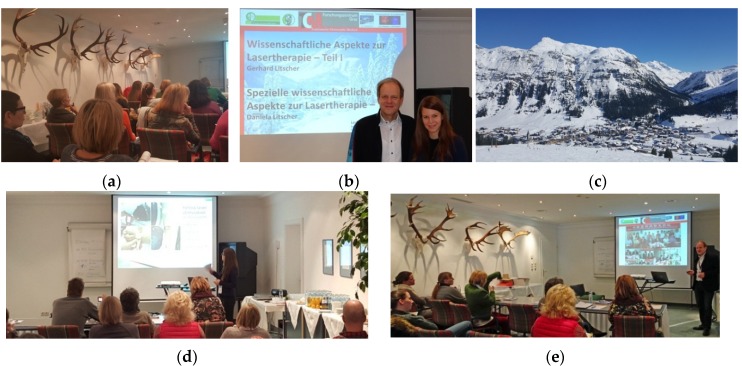
Three-hour seminar (**a**–**e**) from Prof. Gerhard Litscher (**b** left, **e**) and Dr. Daniela Litscher (**b** right, **d**) in Lech (**c**), Austria.

**Figure 3:**
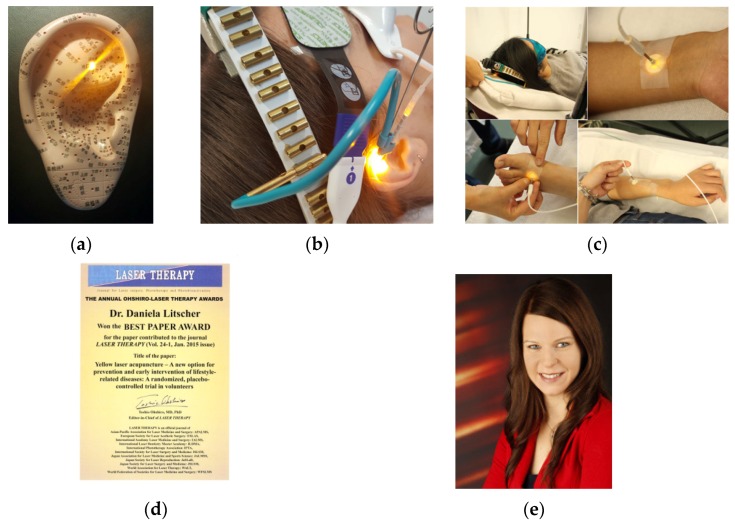
Dr. Daniela Litscher (**e**) receives Ohshiro Laser Therapy Best Paper Award (**d**) for ‘Yellow Laser Acupuncture’ (**a**–**c**).

**Figure 4:**
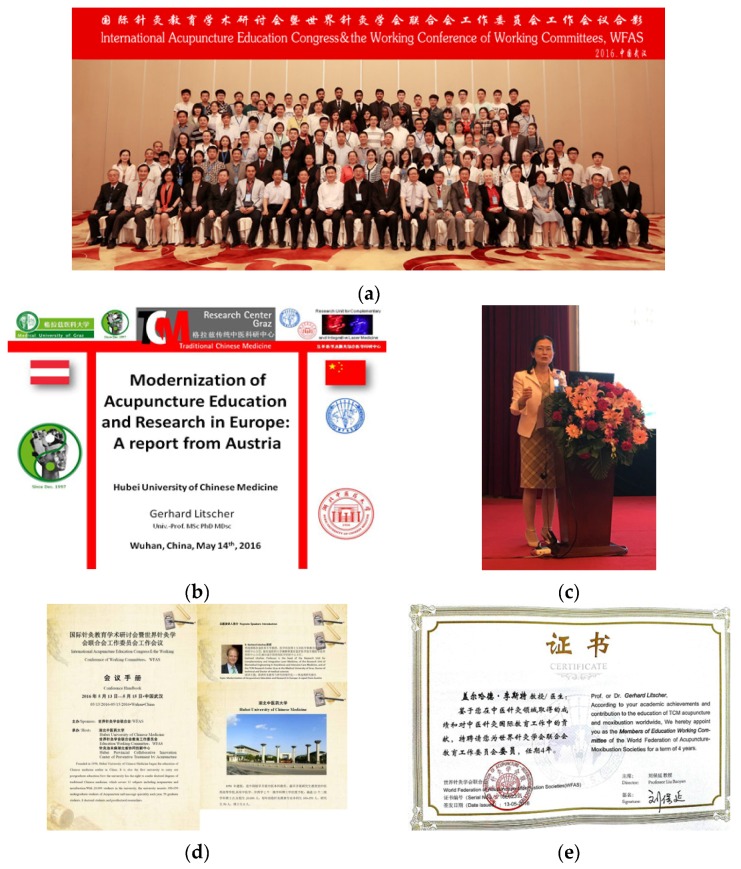
International Acupuncture Education Congress, Wuhan, China (**a**); the lecture from Prof. G. Litscher (**b**) was presented by Prof. Fengxia Liang (**c**). Prof. Gerhard Litscher was appointed as a Member of the Education Working Committee of the World Federation of Acupuncture-Moxibustion Societies for a term of 4 years (**d**,**e**).

**Figure 5:**
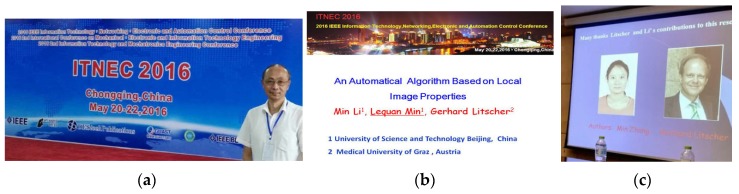
**ITNEC 2016**, Chongqing, China (**a**–**c**); the lecture (**b**,**c**) was presented by Prof. Min Lequan (**a**).

**Figure 6:**
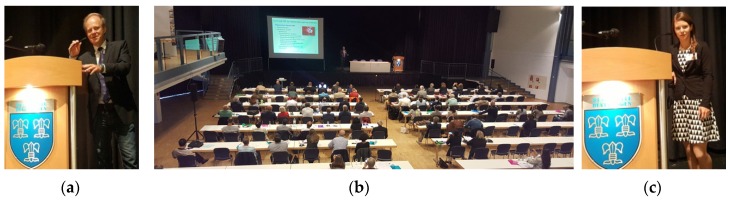
Opening Lecture given by Prof. Gerhard Litscher, President of ISLA (**a**,**b**). Main lecture about Nobel Prize Winner Tu Youyou given by Dr. Daniela Litscher (**c**).

**Figure 7:**
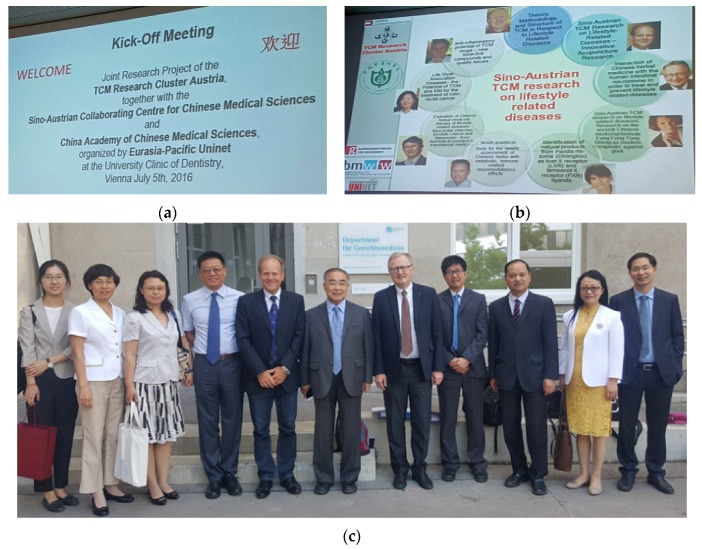
Kick-off Meeting of the joint project (3rd phase) (**a–c**). Professor Zhang Boli (**c** middle) with Prof. Gerhard Litscher (**c** middle left), Prof. Rudolf Bauer (**c** middle left) and the Chinese delegation in Vienna, Austria.

**Figure 8:**
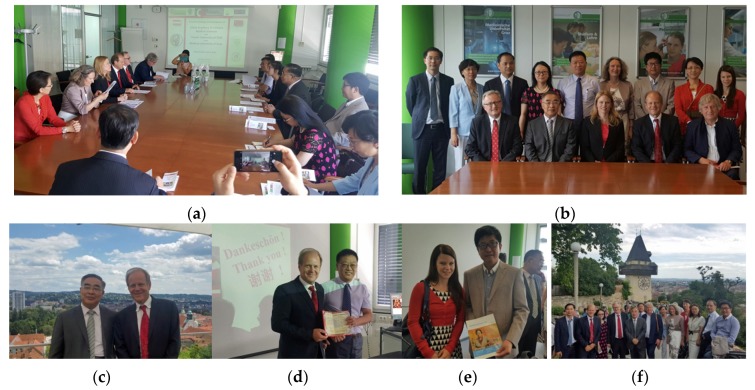
Meeting at Medical University of Graz (**a–f**). Prof. Zhang Boli, President of China Academy of Chinese Medical Sciences and Tianjin University of Traditional Chinese Medicine, and Academician of China Engineering Academy (**c**), Prof. Yu Xiaochun, Executive Deputy Director of Institute of Acupuncture and Moxibustion (**d**), Prof. Chen Shilin, Director of Institute of Chinese Materia Medica (**e**), with Prof. Gerhard Litscher (**c** left) and Dr. Daniela Litscher (**e** right).

**Figure 9:**
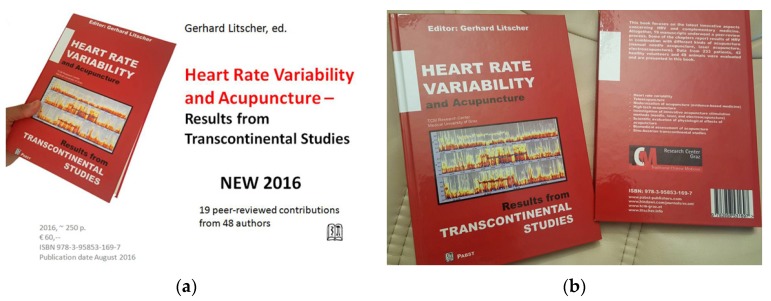
Heart rate variability and acupuncture—results from transcontinental studies (**a**,**b**).

**Figure 10:**
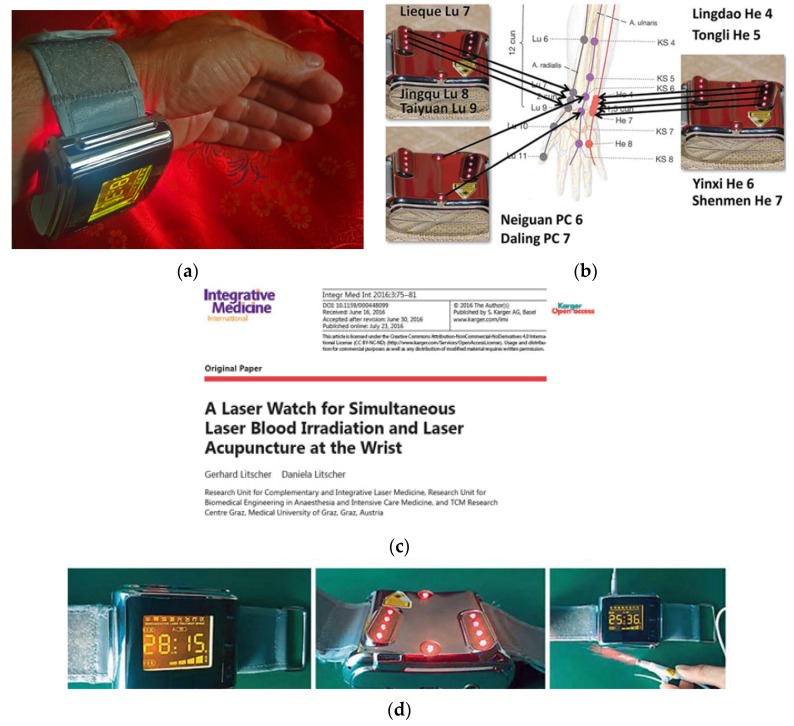
Laser watch (**a**,**d**) for simultaneous laser acupuncture and laser blood irradiation (**b**,**c**) [10].

**Figure 11:**
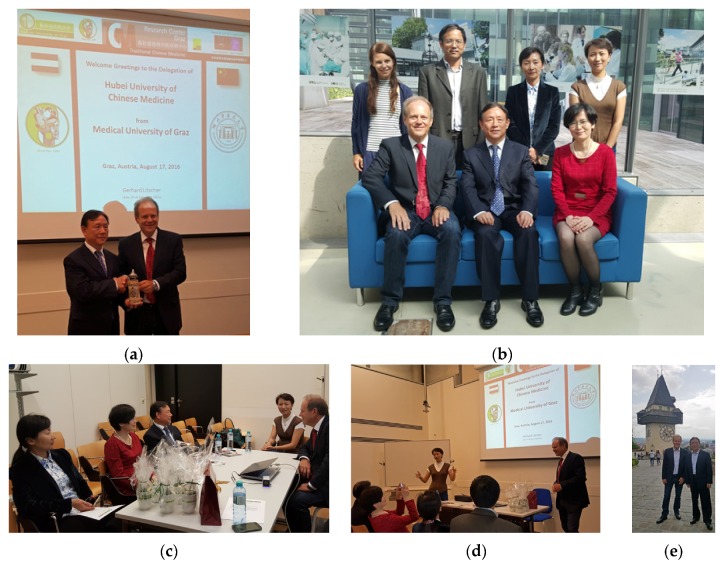
Guests from Hubei University of Chinese Medicine (**a–e**): Prof. Wang Hua, former President of Hubei University of Chinese Medicine (**a** left), Prof. Liang Fengxia, Deputy Director of Acupuncture and Moxibustion Institute (**b** middle), Prof. Qi Fengjun, Acupuncture and Moxibustion and Orthopedics College, Prof. Zhou Zhongyu, Department of Acupuncture and Moxibustion, Traditional Chinese Medicine Hospital of Hubei Province with Prof. Wang Lu, Dr. Daniela Litscher and Prof. Gerhard Litscher.

**Figure 12:**
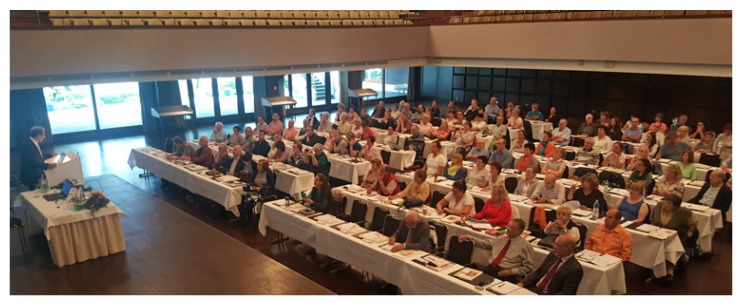
Opening lecture: G. Litscher, 5 September 2016, 8:30–9:30—Innovations and selective trends in the development of ear acupuncture research.

**Figure 13:**
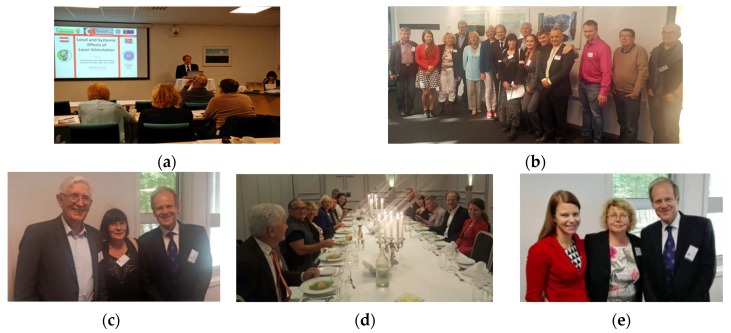
Sandvika Congress (**a–e**). Prof. Juozas V. Vaitkus, President of Lithuanian Physical Society (**c** left), Dr. Anne Harila, President of EMLA Scandinavia (**c** middle), Dr. Daniela Litscher (**e** left), Dr. Draga Marti, President of EMLA Switzerland (**e** middle) and Prof. Gerhard Litscher. Keynote Lecture: G. Litscher (**a**), Local and systemic effects of laser stimulation. Lecture: D. Litscher, Yellow laser: a new option for prevention and treatment of lifestyle-related diseases. Lecture: G. Litscher, Laser watch—a new option for simultaneous laser blood irradiation and laser acupuncture.

**Figure 14:**
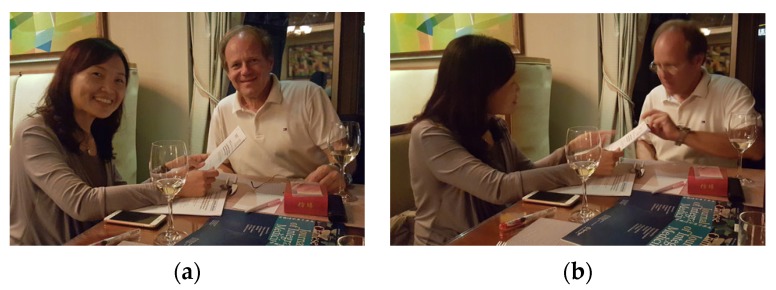
Editorial discussion. Prof. Rong Peijing, Vice director of Auricular Acupuncture Professional Committee, China Association of Acupuncture and Moxibustion (CAAM), Professor at Institute of Acupuncture and Moxibustion at China Academy of Chinese Medical Sciences (**a**,**b** left) and Prof. Gerhard Litscher, Beijing, China, 23 September 2016.

**Figure 15:**
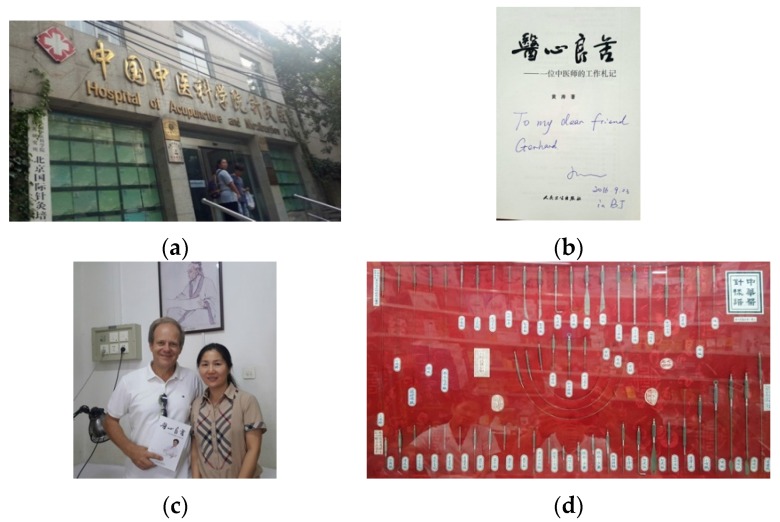
Hospital of Acupuncture and Moxibustion of CACMS (**a**). New book (**b**) from Assoc. Prof. Dr. Huang Tao (**c** right) and Prof. Gerhard Litscher, Beijing, China. Historical acupuncture needles (**d**).

**Figure 16:**
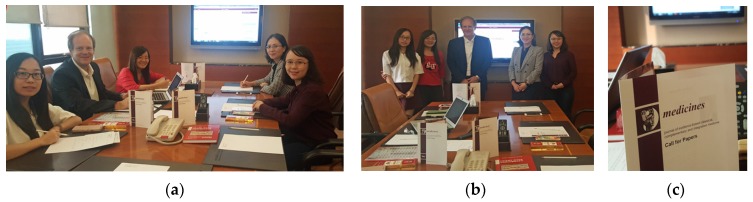
Editor-in-chief Prof. Gerhard Litscher with the editorial team of *Medicines* in Beijing, China. Managing editor Winnie Gong (**a** middle), former managing editor Xiaoyan Chen (**a** left) and assoc. editor-in-chief Prof. Gao Xinyan (**a** second from right).

**Figure 17:**
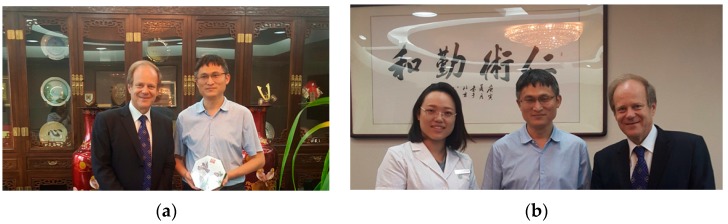

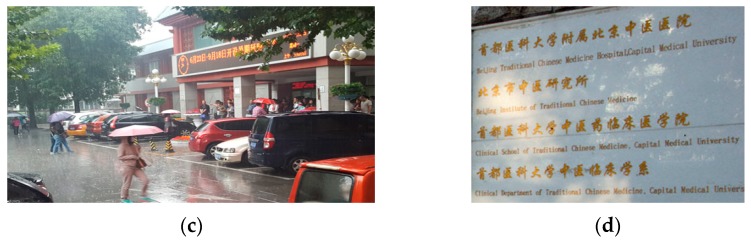
Prof. Liu Cun-Zhi (Vice-Director-China) of the Sino-Austrian Research Center for High-Tech Acupuncture and Clinical and Experimental Integrative Medicine (**a** right, **b** middle) and Prof. Gerhard Litscher (Director of the Center-Austria). Beijing Hospital of TCM (**c**,**d**).

**Figure 18:**
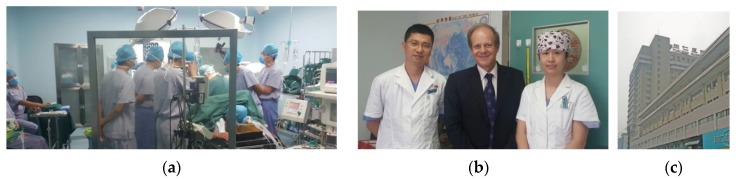
P Tong Ren Hospital (**a**,**c**). Prof. Pan, Head of the Department of Anesthesiology (**b** left), Dr. Sun Yanxia (**b** right) and Prof. Gerhard Litscher (**b** middle).

**Figure 19:**
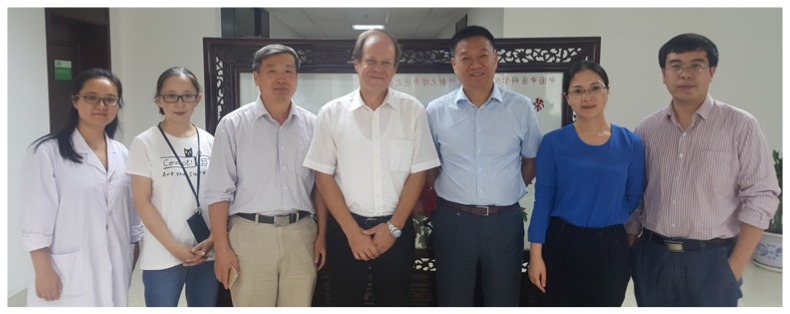
Joint TCM project discussion at CACMS.

**Figure 20:**
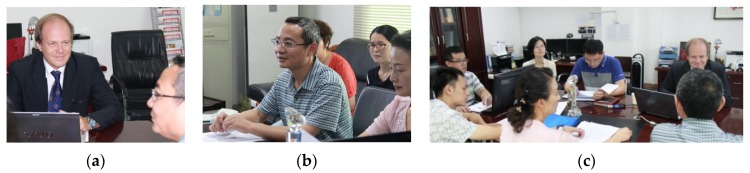
G. Litscher (**a**) Visiting Professor at Hubei University of Chinese Medicine, Wuhan, China, 27 September 2016. Academic discussion with the leaders of Hubei University of Chinese Medicine (**b**,**c**).

**Figure 21:**
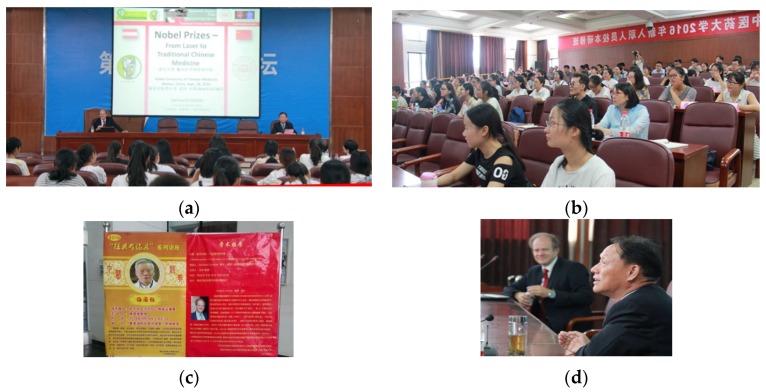
Lecture: G. Litscher (**a**), Nobel Prizes—From Laser to Traditional Chinese Medicine. Prof. Wang Hua, former President of Hubei University of Chinese Medicine (**d**). Lecture room (**b**) and announcement (**c**).

**Figure 22:**
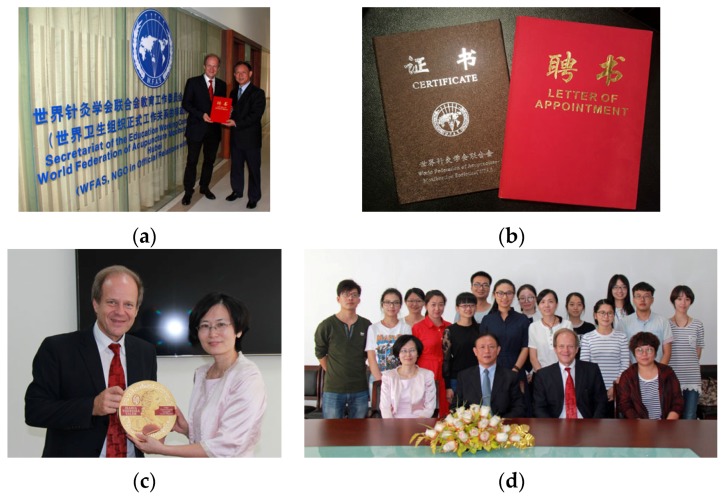
Prof. Gerhard Litscher was appointed as a Member of the Education Working Committee of the World Federation of Acupuncture-Moxibustion Societies in Wuhan/China for a term of 4 years (**a–d**).

**Figure 23:**
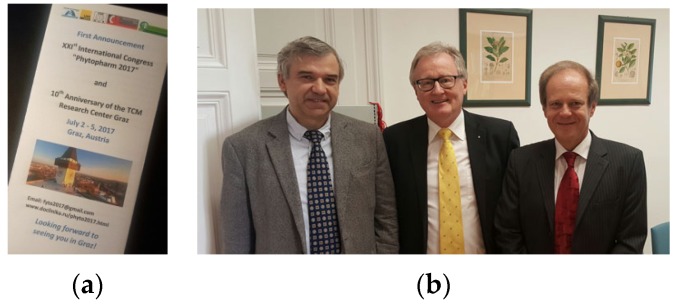
Phytopharm 2017 (**a**). Prof. Alexander Shikov, St. Petersburg, Russia (**b** left), Prof. Rudolf Bauer (**b** middle) and Prof. Gerhard Litscher (**b** right), Graz, Austria (Organizers) [13].

**Figure 24:**
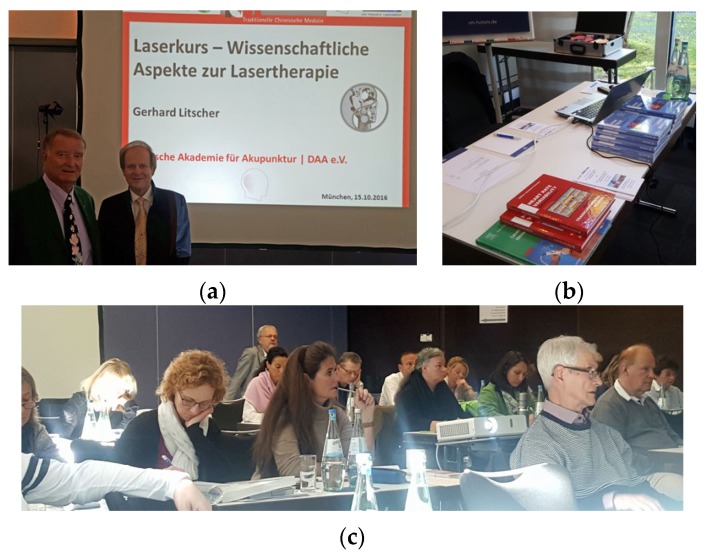
Prof. Frank Bahr, Munich, Germany (**a** left) and Prof. Gerhard Litscher (**a** right), Graz, Austria were the oganizers of the workshop in Munich (**b**,**c**).

**Figure 25:**
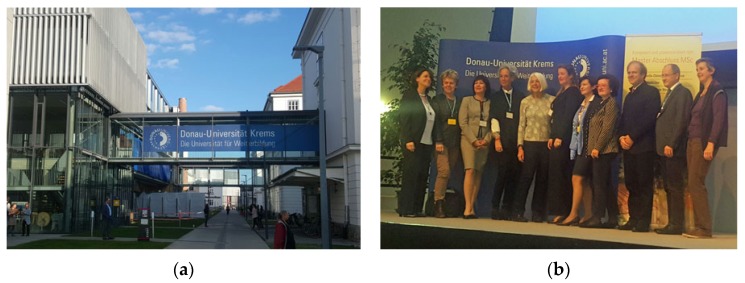
Danube University Krems (**a**) and participants (**b**). Prof. Gerhard Litscher, Lecture + Workshop (Laser Acupuncture and Innovative Laser Medicine).

**Figure 26:**
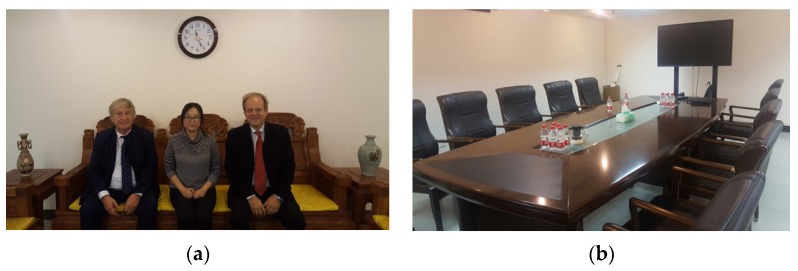
EPU PhD-interviews. Profs. D. Rausch (**a** left) and G. Litscher (**a** right) at Peking University (**b**), Beijing, China.

**Figure 27:**
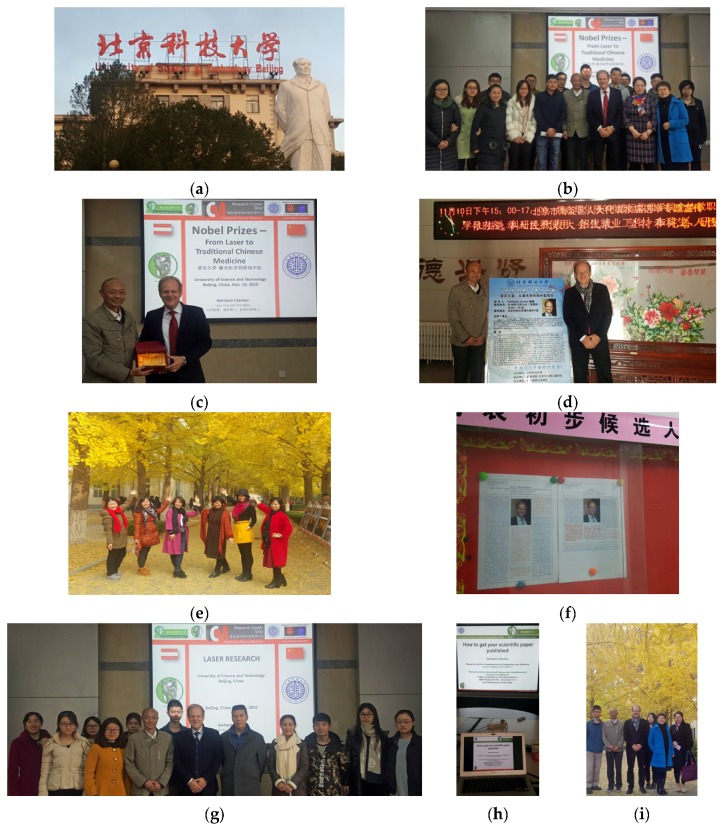
**G** University of Science and Technology Beijing (**a–i**). G. Litscher: lectures as guest professor at USTB (**b–d**,**f–i**), Prof. Min Lequan (**c**,**d** left). Lectures and project discussions.

**Figure 28:**
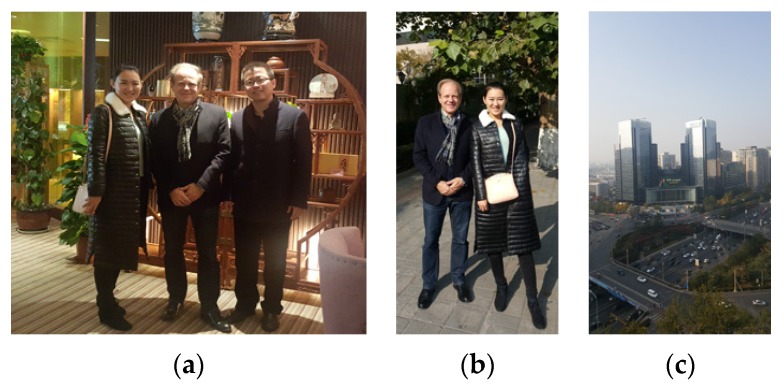
Discussion-clinical study: acupotomy. Prof. Ding Yu (**a** right), Dr. Wang Huan (**a** left, **b** right) and Prof. Gerhard Litscher (**a** middle, **b** left), Beijing (**c**), China.

**Figure 29:**
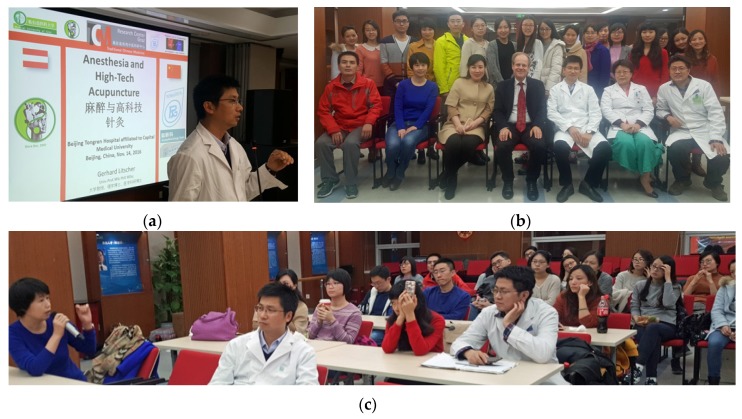
G. Litscher: Lecture-Anesthesia and High-Tech Acupuncture. Prof. Pan, Head of the Department of Anesthesiology (**a**), Dr. Sun Yanxia (**c** left) and Prof. Gerhard Litscher (**b** middle).

**Figure 30:**
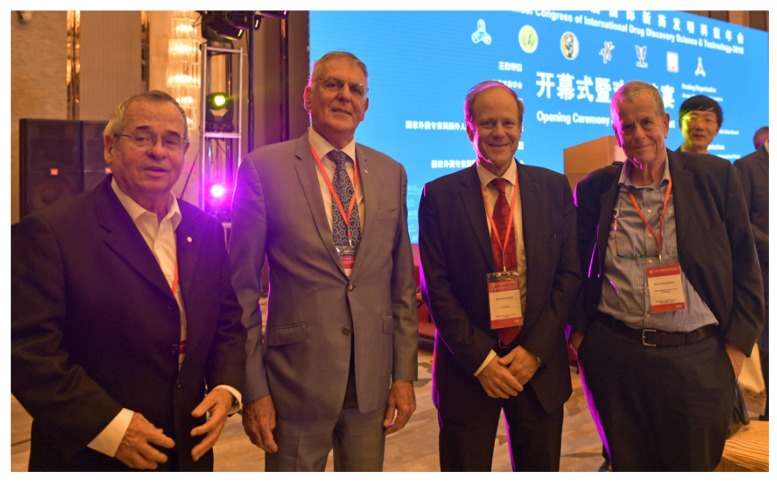
Congress Chair Gerhard Litscher together with three Nobel Prize Winners: Ariel Warshel (left) 2013, Dan Shechtman (middle) 2011 and Aaron Ciechanover (right) 2004.

**Figure 31:**
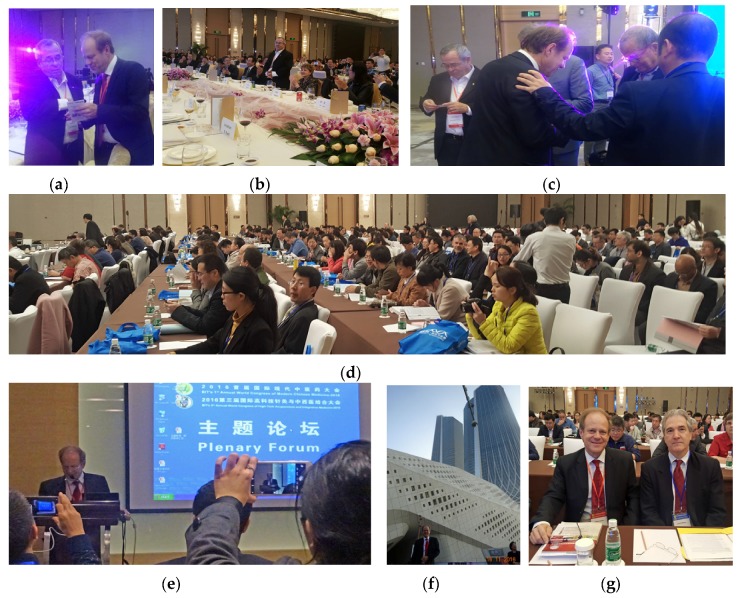

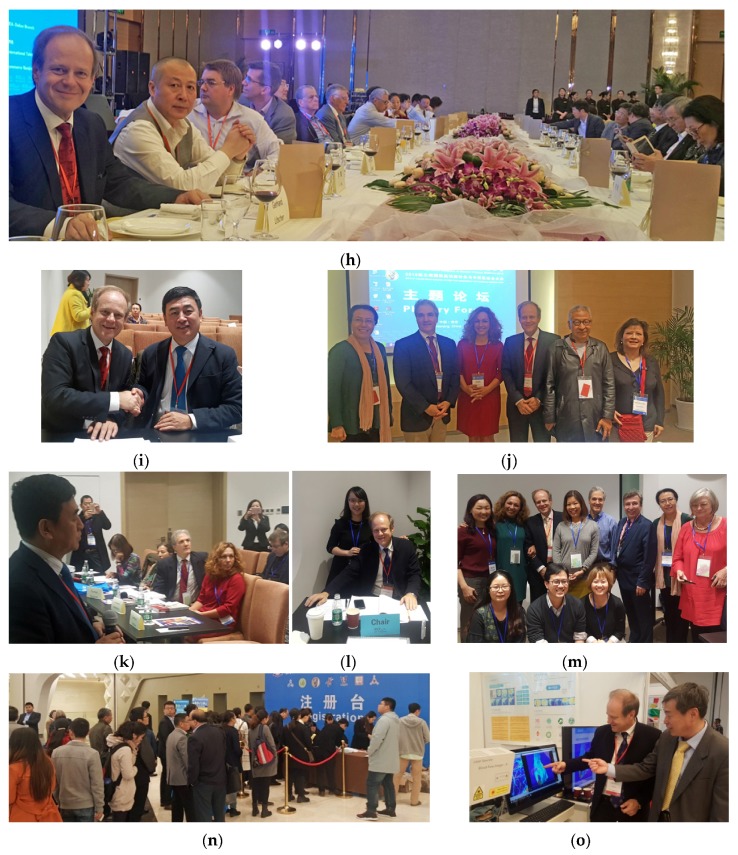
Impressions from the 3rd Annual World Congress High-Tech Acupuncture and Integrative Medicine and the 1st Annual World Congress Modern Chinese Medicine, Nanjing, China (**a–o**).

**Figure 32:**
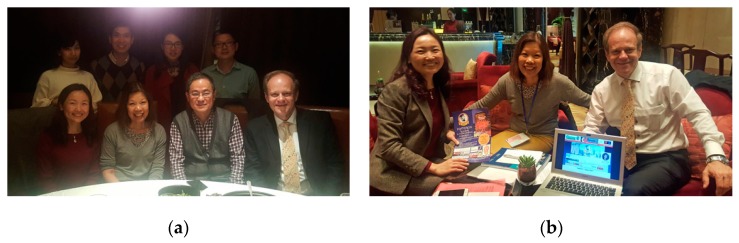
Discussion at Nanjing Medical University. Prof. Rong Peijing, Vice Director of Auricular Acupuncture Professional Committee, China Association of Acupuncture and Moxibustion (CAAM) (**a**,**b** left), Dr. Im Quah Smith, Roseville, Australia, Chair of the Singapore Congress (2017) (**a**,**b** middle) and Prof. Gerhard Litscher (**a**,**b** right). Nanjing, China.

**Figure 33:**
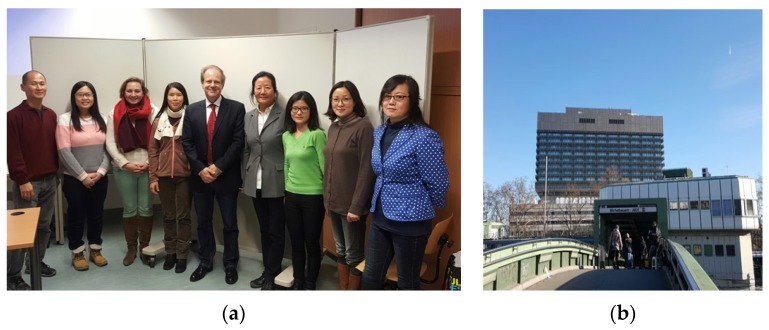
Medical University of Vienna (**b**). Lecture on the topic ‘Experimental Acupuncture’-G. Litscher, Austria. Assoc. Prof. PD Dr. Ma Yan (**a** forth from left), Prof. Gerhard Litscher (**a** middle), and MSc candidates.

**Figure 34:**
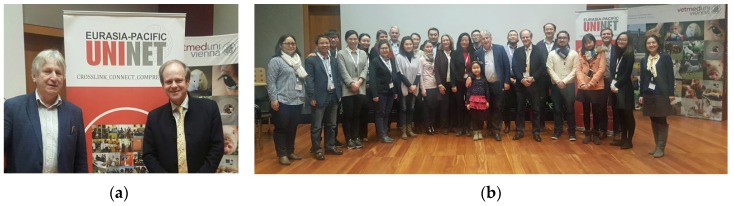
Keynote lecture G. Litscher (**a** right): ‘Exploring Acupuncture within an International High-Tech Network’. EPU network president Prof. W.D. Rausch (**a** left) and EPU scholars (**b**).
